# Supplementary material for: Induction of Chicken Host Defense Peptides within Disease-Resistant and -Susceptible Lines
Source: Genes (Basel). 2020 Oct 14;11(10):1195. doi: 10.3390/genes11101195 (PMC7602260; doi:10.3390/genes11101195)
Supplement: Supplementary file 1 [file genes-11-01195-s001.pdf]

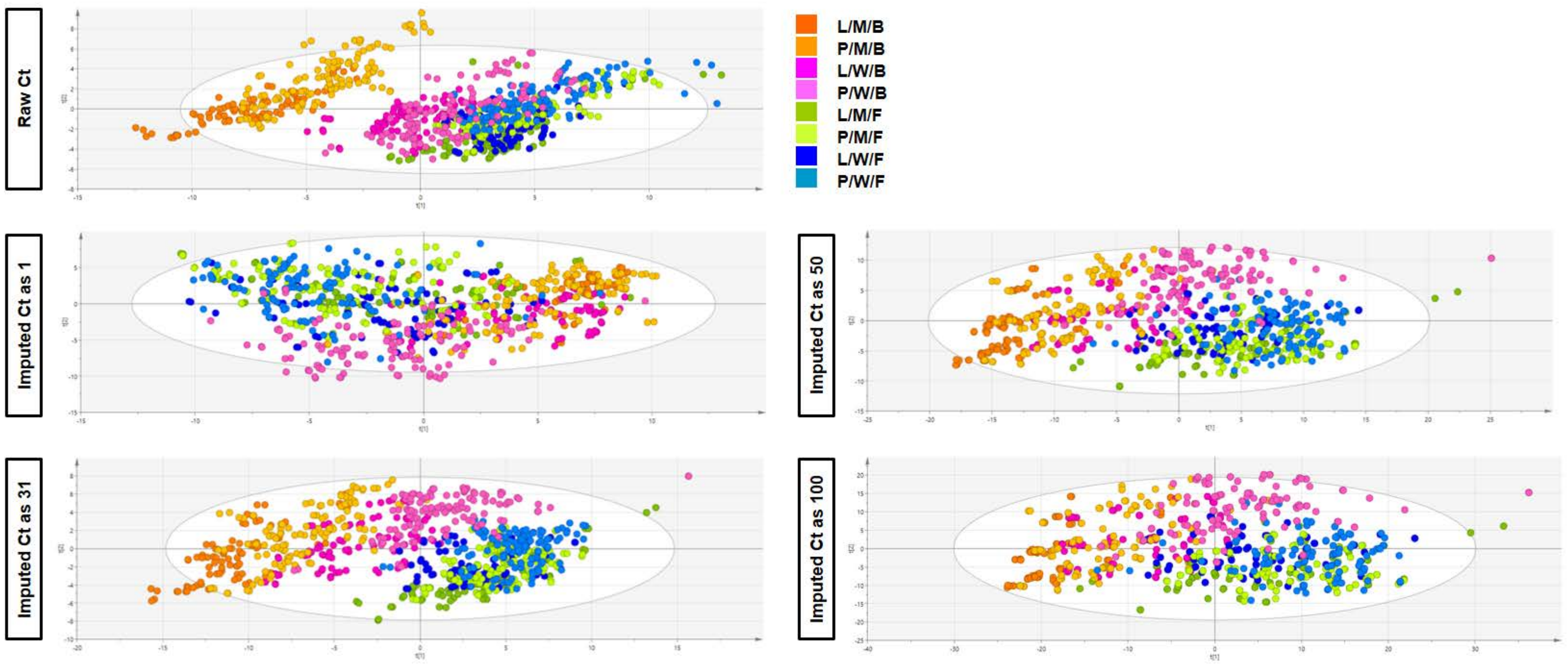

Figure S1. PCA analysis before and after raw Ct imputation. L, LPS-treated experiment; P, poly(I:C)-treated experiment; M, Fayoumi breed; W, Leghorn breed; B, Bone marrow-derived cells; F, Embryonic fibroblasts.

			AvBD1	*AvBD2*	*AvBD3*	*AvBD4*	*AvBD5*	*AvBD6*	*AvBD7*	*AvBD8*	*AvBD9*	*AvBD10*	*AvBD11*	*AvBD12*	*AvBD13*	*AvBD14*	*CATH1*	*CATH2*	*CATH3*	*CATHB1*	*GNLY*	*LEAP2*
NT	BMCs	Line	1.00E-04	1.00E-04	1.00E-04	1.00E-04	1.00E-04	1.25E-02	3.00E-04	1.00E-04	5.00E-04	1.00E-04	3.07E-02	1.30E-03	2.32E-02	5.69E-02	3.29E-01	5.60E-02	4.80E-01	4.00E-04	2.56E-01	1.52E-01
		Lipofection	2.35E-01	1.56E-01	1.72E-01	1.15E-01	3.27E-01	5.53E-01	7.13E-01	8.12E-01	1.30E-01	2.26E-01	2.30E-02	2.77E-01	5.99E-01	2.88E-01	3.65E-01	6.04E-01	3.91E-01	2.26E-01	4.42E-01	2.06E-01
		Time	6.44E-01	5.59E-01	1.85E-01	7.81E-01	4.43E-01	3.14E-01	1.00E+00	7.37E-01	6.59E-01	6.94E-01	1.39E-01	2.17E-01	1.00E-02	5.37E-01	4.22E-01	4.47E-01	8.65E-01	9.88E-01	4.08E-01	9.85E-02
	CEF	Line	4.81E-01	2.47E-01	5.90E-02	1.57E-01	1.00E-04	9.15E-01	6.15E-01	1.43E-02	1.57E-02	3.23E-02	8.50E-03	1.00E-04	3.50E-03	7.12E-01	2.00E-04	2.11E-01	2.65E-02	1.00E-04	1.00E-04	5.91E-02
		Lipofection	8.12E-01	7.78E-01	2.88E-01	8.18E-02	6.30E-01	4.25E-01	4.79E-01	7.57E-01	5.17E-01	9.66E-01	9.57E-02	7.96E-02	9.33E-02	3.92E-02	8.10E-03	7.03E-01	7.39E-01	8.38E-01	5.90E-01	1.08E-01
		Time	9.18E-01	5.84E-01	1.23E-01	6.03E-01	9.81E-02	7.33E-01	2.65E-01	3.49E-01	4.46E-01	7.94E-01	4.31E-01	3.65E-01	8.19E-01	9.39E-01	1.54E-01	8.30E-01	1.00E-04	6.73E-01	3.70E-03	2.30E-03
LPS	BMCs	Line	1.00E-04	1.00E-04	1.00E-04	1.00E-04	1.00E-04	4.84E-02	1.20E-02	1.61E-01	4.60E-03	7.70E-03	4.51E-01	8.32E-01	4.29E-01	1.00E-04	1.00E-04	1.44E-02	1.23E-01	1.11E-02	1.00E-04	2.60E-03
		Treatment	2.70E-03	1.00E-04	4.84E-02	4.70E-03	1.06E-02	6.40E-01	8.00E-01	8.75E-01	1.61E-02	5.47E-01	4.84E-01	8.40E-03	1.18E-01	1.90E-01	2.08E-02	2.88E-01	5.48E-02	1.00E-04	2.63E-01	5.34E-01
		Time[Treatment]	1.90E-03	1.00E-04	3.93E-01	7.00E-04	4.78E-01	9.66E-01	9.84E-01	9.07E-01	1.00E-04	6.83E-01	7.32E-02	1.90E-03	7.84E-01	2.38E-01	4.37E-01	3.03E-01	3.56E-01	1.00E-04	2.82E-01	3.93E-01
		Treatment*Line	5.13E-01	5.30E-03	4.21E-02	5.76E-01	3.41E-02	3.55E-01	9.66E-01	1.65E-01	5.71E-02	5.91E-01	8.84E-01	7.74E-02	1.96E-01	1.32E-01	5.31E-01	9.21E-01	4.11E-02	1.00E-04	4.37E-01	2.39E-01
		Time*Line[Treatment]	3.13E-02	5.34E-01	5.47E-01	6.52E-01	8.42E-01	1.10E-01	2.25E-01	5.80E-01	1.03E-02	6.33E-01	4.84E-02	4.13E-01	1.02E-01	8.91E-01	9.98E-01	5.69E-01	2.90E-02	1.00E-04	3.26E-01	9.20E-01
	CEFs	Line	3.80E-01	7.15E-01	5.30E-01	7.17E-01	1.52E-02	8.84E-01	4.13E-01	5.48E-01	4.54E-01	2.18E-01	7.18E-01	6.70E-03	3.51E-02	3.35E-01	6.59E-01	8.96E-01	5.99E-02	1.00E-04	3.88E-01	9.75E-01
		Treatment	2.62E-01	9.93E-01	4.43E-01	2.19E-01	2.32E-01	9.20E-01	2.19E-01	4.45E-01	4.68E-01	8.11E-01	7.85E-01	6.33E-01	8.32E-01	2.34E-01	7.80E-01	4.95E-01	1.14E-01	4.07E-01	7.91E-01	4.02E-01
		Time[Treatment]	8.65E-01	8.11E-01	2.00E-02	3.08E-01	7.05E-01	3.68E-01	7.42E-01	6.51E-01	7.37E-01	8.69E-01	3.87E-01	1.01E-01	1.63E-01	8.20E-01	8.55E-01	3.86E-01	6.54E-01	2.04E-01	8.02E-01	8.57E-01
		Treatment*Line	4.48E-01	7.50E-01	3.17E-01	8.85E-01	1.97E-01	7.44E-01	5.09E-01	3.96E-01	3.49E-01	7.27E-01	5.77E-01	7.93E-01	1.17E-01	3.61E-01	7.65E-01	6.84E-01	8.03E-01	8.12E-01	7.26E-01	9.26E-01
		Time*Line[Treatment]	8.11E-01	9.19E-01	2.24E-01	8.93E-01	6.21E-01	2.34E-01	5.47E-01	6.13E-01	5.52E-01	8.37E-01	3.91E-01	6.94E-01	8.78E-01	3.99E-01	3.58E-01	5.33E-01	6.05E-01	7.91E-01	3.06E-01	3.84E-01
Poly(I:C)	BMCs	Line	1.00E-04	1.00E-04	1.00E-04	1.00E-04	1.00E-04	1.00E-04	1.00E-04	1.00E-04	1.00E-04	1.00E-04	1.00E-04	1.00E-04	1.30E-03	1.14E-02	3.58E-01	1.45E-02	6.45E-01	1.00E-04	2.34E-01	3.97E-02
		Treatment	5.87E-01	2.70E-03	3.47E-01	9.73E-01	4.50E-01	1.43E-01	6.52E-01	2.36E-01	6.28E-01	3.63E-01	1.44E-02	1.00E-04	2.60E-03	3.03E-01	1.64E-01	3.75E-02	2.95E-01	3.60E-03	1.92E-01	4.28E-02
		Time[Treatment]	5.99E-01	1.54E-01	1.47E-01	9.07E-01	4.83E-01	5.77E-01	9.89E-01	3.88E-01	5.15E-01	8.80E-01	4.36E-02	2.42E-02	7.50E-02	3.85E-01	3.81E-01	3.56E-01	3.99E-01	2.10E-03	3.25E-01	4.15E-02
		Treatment*Line	5.65E-01	3.58E-01	5.57E-01	9.05E-01	6.86E-01	8.94E-01	8.85E-01	6.00E-01	6.73E-01	7.81E-01	1.55E-01	2.22E-01	5.23E-01	4.40E-01	4.85E-01	5.47E-01	5.54E-01	8.08E-01	4.17E-01	6.60E-01
		Time*Line[Treatment]	4.34E-01	1.29E-01	2.20E-02	8.68E-01	1.57E-01	1.69E-01	8.83E-01	3.59E-01	4.96E-01	3.45E-01	1.75E-01	1.55E-01	4.88E-01	3.06E-01	2.73E-01	2.01E-01	2.50E-01	9.69E-01	3.52E-01	2.55E-01
	CEFs	Line	3.83E-01	8.08E-01	5.05E-02	1.25E-01	1.00E-04	3.88E-01	1.37E-02	1.60E-03	1.78E-02	2.13E-02	1.11E-02	1.00E-04	6.10E-03	9.42E-01	1.70E-03	1.32E-02	1.91E-02	1.00E-04	1.00E-04	3.39E-02
		Treatment	2.53E-01	1.00E-04	2.83E-02	6.57E-01	1.22E-01	4.20E-02	6.51E-02	5.76E-01	1.83E-02	1.37E-01	4.40E-03	1.00E-04	3.73E-02	1.01E-02	1.00E-04	7.35E-02	4.00E-04	1.00E-04	1.00E-04	1.00E-04
		Time[Treatment]	5.45E-01	4.50E-03	4.67E-01	3.49E-01	3.64E-01	6.22E-01	1.89E-01	2.21E-01	3.04E-01	9.20E-01	1.73E-01	9.50E-03	9.86E-01	1.47E-02	1.00E-04	4.70E-03	1.00E-04	1.00E-04	1.00E-04	7.60E-03
		Treatment*Line	7.61E-02	4.48E-01	8.87E-01	3.29E-01	4.06E-01	4.13E-01	2.88E-02	8.20E-01	9.84E-01	6.28E-01	9.46E-01	1.43E-01	1.68E-01	9.47E-01	6.61E-02	6.08E-01	4.01E-01	9.29E-01	1.32E-01	6.02E-01
		Time*Line[Treatment]	9.90E-01	3.00E-02	8.69E-01	5.47E-01	8.66E-01	5.25E-01	8.52E-01	4.99E-01	8.40E-01	6.00E-01	1.75E-01	6.30E-02	3.79E-01	3.93E-01	1.69E-01	3.54E-01	4.80E-02	8.50E-03	5.61E-01	3.63E-01

Figure S2. Statistical analysis of dCt values of host defense peptide (HDP) genes among experimental groups. P-values for the fixed effects in each linear regression model (for NT, LPS and poly(I:C)) are shown. NT, non-treated; LPS, lipopolysaccharide; poly(I:C), polyinosinic:polycytidylic acid; BMC, bone marrow-derived cells; CEF, chicken embryonic fibroblasts.

(yellow), $P < 0.05$; (orange), $P < 0.01$; (red), $P < 0.001$.
